# Special Issue “Cancer Biomarker Research and Personalized Medicine”

**DOI:** 10.3390/jpm12040585

**Published:** 2022-04-05

**Authors:** James Meehan

**Affiliations:** Translational Oncology Research Group, Institute of Genetics and Cancer, Western General Hospital, University of Edinburgh, Edinburgh EH4 2XU, UK; jmeehan@ed.ac.uk

While the term biomarker is thought to have first been used in the 1970s, the concept itself is considered to be much older. The turn of the 21st century saw a dramatic increase in the number of papers published concerning biomarkers. Biomarkers can be described as characteristics that can be assessed and quantified as indicators of standard biological processes, pathogenesis or response to therapy. The treatment of individual patients based on particular factors, such as biomarkers, distinguishes standard, generalized treatment plans from personalized medicine. Even though personalized medicine is applicable to most branches of medicine, the field of oncology is perhaps where it is most easily employed. Cancer is a heterogeneous disease; although patients may be diagnosed histologically with the same cancer type, their tumors can comprise varying tumor microenvironments and molecular characteristics that can impact treatment response and prognosis.

There has been a major drive over the past decade to try and realize personalized cancer medicine through the discovery and use of disease-specific biomarkers. This Special Issue, entitled “Cancer Biomarker Research and Personalized Medicine”, encompasses 22 publications from colleagues working on a diverse range of cancers, including prostate, breast, ovarian, head and neck, liver, gastric, bladder, colorectal and kidney. The biomarkers assessed in these studies include genes, intracellular or secreted proteins, exosomes, DNA, RNA, miRNA, circulating tumor cells and circulating immune cells, in addition to radiomic features.

A number of different biomarker subtypes have been delineated according to their recognized applications. Biomarkers can be defined by the mechanisms that lead to disease development, perhaps linked with susceptibility/risk factors that can initiate a pathophysiological process. Susceptibility/risk biomarkers reveal the possibility for developing a disease in those that do not currently have a clinically apparent disease. Work submitted to this Special Issue highlights the potential of DNA methylation as a risk biomarker for head and neck cancer [1].

Diagnostic biomarkers differ in that they detect/confirm the presence of a disease. The early diagnosis of cancer is vital for improving the survival of patients. Several publications within this Special Issue explore diagnostic biomarkers, with research providing new insights into the development of diagnostic biomarkers for prostate cancer [2], hepatocellular carcinoma [3], gastric cancer [4] and head and neck cancer [5]. As we progress further into the precision medicine era, diagnostic biomarkers will continue to evolve; such biomarkers may not only be utilized to identify those with cancer, but also to re-define the classification of cancer [6].

In patients that have already been diagnosed with cancer, it can be challenging to stratify those with tumors that are less likely to progress from patients with tumors that are more aggressive and therefore require treatment intensification; tumor heterogeneity contributes greatly to this problem. While the early diagnosis of cancer is crucial for enhancing the survival of patients, the identification of biomarkers at the time of diagnosis that can give an indication of cancer aggressiveness is possibly the greatest unmet clinical need for many cancer types [2,7]. Prognostic biomarkers identify the likelihood of disease recurrence or progression; these factors are crucial to decision-making processes in the clinic, helping clinicians determine the most appropriate treatment for each patient. Several publications within this Special Issue explored prognostic biomarkers, focusing on the development of prognostic tissue-based biomarkers in ovarian cancer [8], prostate cancer [2,7] and renal cell carcinoma [9], in addition to prognostic liquid-based biomarkers in prostate cancer [2], bladder cancer [10] and head and neck cancer [11]. Studies published within the Special Issue also show how cancer prognosis is moving towards the use of imaging, rather than relying on tissue/liquid biomarkers alone [12].

While prognostic biomarkers can help identify patients at a higher risk who might benefit from more aggressive treatment, they do not give any information on which patients are likely to gain a clinical benefit from a specific therapy. Conversely, predictive biomarkers are those that can indicate the probability of a patient gaining a therapeutic benefit from a specific treatment. While many of the standard cancer treatments such as radiotherapy and chemotherapy are effective, the use of these treatments in non-responding patients is associated with increased levels of toxicity and can delay the of instigation of alternative treatments that may have a greater effect. As such, predictive biomarkers represent a major research area, with work submitted to this Special Issue showing their potential to predict response to chemotherapy [13,14], radiotherapy [15], chemo-radiotherapy [16] and therapeutic cancer vaccines [17] in various tumor types.

Monitoring biomarkers are those that can be measured serially to evaluate the status of a disease or to assess treatment response. These types of biomarkers are useful to detect evidence of early therapeutic response, or to reveal complications resulting from a therapy [2]. Although biomarkers have been defined according to specific applications, biomarkers may also meet multiple criteria for different uses. Numerous papers contributed to this Special Issue deal with biomarkers that fall into this category, including studies involving colorectal [18] and breast [19,20,21] cancers.

Cancer biomarker research, translated from the lab to the clinic, has led to a significant improvement in patient management, leading to increased survival rates and improved quality of life, while also lowering healthcare costs. Additional research into the detection of novel mutational variants to identify genes that are driving cancer development is critical for biomarker discovery and the further development of personalized medicine [22]. Detailed genotypic/phenotypic evaluation of individual patients is becoming increasingly available and is occurring in tandem with the development of new and improved treatments. Further advances will allow personalized medicine to become a reality for all cancer types in the decades to come.

## Data Availability

Not applicable.
